# Body surface assessment with 3D laser-based anthropometry: reliability, validation, and improvement of empirical surface formulae

**DOI:** 10.1007/s00421-016-3525-5

**Published:** 2017-01-27

**Authors:** Andreas Kuehnapfel, Peter Ahnert, Markus Loeffler, Markus Scholz

**Affiliations:** 1 Leipzig Research Center for Civilization Diseases (LIFE), University of Leipzig, Leipzig, Germany; 2 Institute for Medical Informatics, Statistics and Epidemiology (IMISE), University of Leipzig, Leipzig, Germany

**Keywords:** 3D body scanner, Anthropometry, Body surface area, Reliability, Validity

## Abstract

**Purpose:**

Body surface area is a physiological quantity relevant for many medical applications. In clinical practice, it is determined by empirical formulae. 3D laser-based anthropometry provides an easy and effective way to measure body surface area but is not ubiquitously available. We used data from laser-based anthropometry from a population-based study to assess validity of published and commonly used empirical formulae.

**Methods:**

We performed a large population-based study on adults collecting classical anthropometric measurements and 3D body surface assessments (*N* = 1435). We determined reliability of the 3D body surface assessment and validity of 18 different empirical formulae proposed in the literature. The performance of these formulae is studied in subsets of sex and BMI. Finally, improvements of parameter settings of formulae and adjustments for sex and BMI were considered.

**Results:**

3D body surface measurements show excellent intra- and inter-rater reliability of 0.998 (overall concordance correlation coefficient, OCCC was used as measure of agreement). Empirical formulae of Fujimoto and Watanabe, Shuter and Aslani and Sendroy and Cecchini performed best with excellent concordance with OCCC > 0.949 even in subgroups of sex and BMI. Re-parametrization of formulae and adjustment for sex and BMI slightly improved results.

**Conclusion:**

In adults, 3D laser-based body surface assessment is a reliable alternative to estimation by empirical formulae. However, there are empirical formulae showing excellent results even in subgroups of sex and BMI with only little room for improvement.

**Electronic supplementary material:**

The online version of this article (doi:10.1007/s00421-016-3525-5) contains supplementary material, which is available to authorized users.

## Introduction

Body surface area is a physiological quantity relevant for many medical applications. First, it serves as a measure of standardization, e.g., for echocardiographic assessment or dosage of cytotoxic and cytostatic drugs in cancer therapy (Baker et al. 2002; Pinkel 1958). For the latter, it is believed that body surface area correlates with size and function of drug-metabolizing organs, but this is also criticized in the literature by others (Gurney 1996, 2002). Another important area is assessment of the severity of skin lesions, e.g., in case of burnings (Scarisbrick and Morris 2013). The evolutionary development of body surface area/body weight ratio was investigated for different climate regions in several studies finding a positive correlation with temperature (Katzmarzyk and Leonard 1998; Roberts 1953; Ruff 1991, 1994; Wheeler 1984).

Due to the complex geometry of the human body, it is not easy to determine body surface area. Empirical formulae have been suggested for this purpose, e.g., DuBois and DuBois (1989), Haycock et al. (1978) and Mosteller (1987). The first proposed equation to estimate the surface of the human body was derived by Meeh (1879). His formula includes only body weight as the variable for prediction. Later on until today, almost all available and reliable formulae are based on non-linear functions of body height and body weight. Often used techniques to provide a data base for derivation of formulae were coating (Shuter and Aslani 2000), surface integration (e.g. Anderson 1985), and triangulation (Gehan and George 1970). Since these measurement techniques are demanding, there are typically small data sets. Thus, statistical inference of model parameters is limited, especially considering applicability to different groups of individuals, such as children, males, females, or extreme builds.

Laser-based 3D anthropometric assessments offer a way for easy and effective measurement of body surface area allowing collection of large data sets. By this procedure, probands are scanned with lasers from four directions. This results in a “virtual twin” generated by optical triangulation. The point cloud of the virtual twin can later be subjected to biometric analyses eventually resulting in a number of anthropometric measurements, including the body surface area. After determining the reliability of this assessment on the basis of a small study of repeated measurements (Loeffler et al. 2015; Kuehnapfel et al. 2016), we analyzed a large data set of healthy adults collected in the framework of an epidemiologic study to verify available empirical formulae. Moreover, we re-estimate the parameters of empirical formulae for possible improvements. Finally, we studied the performance of the formulae for males and females separately and for different BMI categories.

## Methods

### Study

Data are available from the population-based LIFE-Adult study, which recruited 10,000 adults from the city of Leipzig, Germany. The aim of the study is to analyze lifestyle, environmental and molecular genetic risk factors of civilization diseases with emphasis on metabolic and cardiovascular disorders, depression and cognitive impairment. Details of the study can be found elsewhere (Loeffler et al. 2015). All subjects included in the study gave written informed consent. The study was approved by the ethics committee of the Medical Faculty of the University of Leipzig (263-2009-14122009) and was performed adhering to the principles of the Declaration of Helsinki.

### Classical anthropometry and body surface area determined by formulae

Classical anthropometric assessments were performed for almost all LIFE participants allowing us to apply empirical formulae for body surface area. Anthropometric measurements of body height and body weight were performed at room temperature of about 22 °C. Body height was determined with a stadiometer (seca 220, seca, Hamburg, Germany), while body weight was ascertained with a body scale (seca 701, seca, Hamburg, Germany). Subjects were instructed to stand upright and stretched, heels kept close to each other and legs straightened. Weight had to be placed uniformly on both feet. Arms and hands hang down loose and had to be stretched.

We applied a total of 18 empirical formulae obtained from the literature to calculate the body surface area. Surface formulae are chosen due to their occurrence and citation in practical applications. Formulae typically require height, weight, or both of them (cf. Table 1).

**Table 1:** Body surface area equations for comparison with 3D body scans

Name of surface formula	Formula	Reference
Anderson	0.0239 × height^0.417^ × weight^0.517^	Anderson (1985)
Bardeen	0.000143 × (2 × 1000/height × weight + 4 × height × (1000/height × weight)^0.5^)	Bardeen (1920)
Boyd	0.01787 × height^0.5^ × weight^0.4838^	Boyd (1935)
Brody	0.02411 × height^0.4^ × weight^0.53^	Brody (1945)
DuBois and DuBois	0.007184 × height^0.725^ × weight^0.425^	Du Bois and Du Bois (1989)
Fujimoto and Watanabe	0.008883 × height^0.663^ × weight^0.444^	Fujimoto and Watanabe (1969)
Gehan and George	0.0235 × height^0.42246^ × weight^0.51456^	Gehan and George (1970)
Haycock et al.	0.024265 × height^0.3964^ × weight^0.5378^	Haycock et al. (1978)
Isaksson	1 + (|height − 160| + weight)/100	Isaksson (1958)
Livingston and Lee	0.1173 × weight^0.6466^	Livingston and Lee (2001)
Mosteller	0.0167 × height^0.5^ × weight^0.5^	Mosteller (1987)
Reading and Freeman	height^0.5^/60 × weight^0.5^	Reading and Freeman (2005)
Schlich et al.	Male: 0.000579479 × height^1.24^ × weight^0.38^ Female: 0.000975482 × height^1.08^ × weight^0.46^	Schlich et al. (2010)
Sendroy and Cecchini	0.0097 × (height + weight) − 0.545	Sendroy and Cecchini (1954)
Shuter and Aslani	0.00949 × height^0.655^ × weight^0.441^	Shuter and Aslani (2000)
Takahira	0.007241 × height^0.725^ × weight^0.425^	Fujimoto and Watanabe (1969)
Tikuisis et al.	Male: 0.0128 × height^0.6^ × weight^0.44^ Female: 0.0147 × height^0.55^ × weight^0.47^	Tikuisis et al. (2001)
Wang and Hihara	0.0168 × height^0.5^ × weight^0.5^	Wang and Hihara (2004)

Height in cm. Weight in kg. Resulting surface in m^2^

### Body surface area determined by body scanner software

Almost all participants received 3D anthropometric scan. Bodyscanner measurements were performed at room temperature of about 22 °C. ANTHROSCAN VITUS XXL SYSTEM comprising 3D Body Scanner VITUS XXL with the ANTHROSCAN BASIS software (version 3.0.1, laser class 1—safe with open eyes, Human Solutions, Kaiserslautern, Germany) was used for 3D anthropometry.

Probands were required to wear only tightly fitting underpants, stockings, and bras (women). Underwear should not be of dark color not to disturb the laser scan. One-way underwear was available for this purpose if required. Exceptions were documented and considered during analysis by visual inspection of the 3D body scan. If necessary, hair accessories and jewelry should be removed if possible. For correct height measurement, individuals were asked to wear a tight fitting bathing cap. Longer hair was required to be hidden under the bathing cap without substantially changing the shape of the head. The seventh neck vertebra had to be exposed and ears had to be uncovered. Individuals were advised to set their feet shoulder-wide apart on the marked areas of the scanner platform. Thighs should not touch below the crotch, if possible. Weight should be distributed equally on both legs. Participants were asked to bring themselves in an upright and relaxed posture, standing as naturally as possible. Arms had to be slightly spread, and elbows slightly bent. Individuals were asked to make fists, with thumbs outside, pointing forward. Eyes should be kept open while scanning, fixing a point on the wall at eye level, and not following the laser beam. Between repeated measurements, subjects were asked to step off the scanner. Instructions for positioning on the scanner platform were repeated for each scan.

The software automatically derives 154 anthropometric measures from each 3D scan. After scanning the proband, a virtual twin consisting of 500,000 data points on average is obtained by optical triangulation. The body scanner software automatically determines implemented anthropometric measurements on the basis of DIN EN ISO 20685 with the possibility to perform manual adjustments. A quality check is made by visual inspection of the scanning image. However, body surface area had to be calculated separately, since this routine was not implemented in the standard software. Software version (version 3.0.1) was used to determine the body surface area. Since batch analysis was not implemented, this step required manual upload of body scanner files which is time-consuming. Therefore, we determined body surface area for a consecutive subset of LIFE-Adult.

Regarding validation of the software, one has to acknowledge that the algorithm and the implemented source code are not available. Comparison of body scanner-derived body surface area and direct measurements of the surface, e.g., using body coating, was not feasible and we are not aware of any published data with respect to that issue. However, we randomly compared body surface area obtained by the proprietary software with results from the standard mathematical software (Mathematica) resulting in highly similar values.

### Statistical analyses

For a total of 1435 participants, both classical and 3D derived surface areas are available. These data were used for verification of the empirical formula. Sex and BMI distribution of these 1435 individuals are similar to the entire study sample of LIFE-Adult.

In a separate data set of 126 subjects, two body scans were performed in the framework of a feasibility study (Kuehnapfel et al. 2016) in preparation for the LIFE-Adult study. For each of them, body surface area was determined. These data were used to assess the reliability of the 3D-based body surface calculation. The study design (four study arms: two for intra- and inter-rater reliability of classical anthropometry and two for intra- and inter-rater reliability of laser-based anthropometry) allows calculating both intra- and inter-rater reliabilities. The term “intra-rater reliability” refers to the comparison of two measurements of those 67 participants for which the second measurement was taken by the same examiner. In contrast, “inter-rater reliability” could be estimated on the basis of 59 participants that were the two measurements that were taken by two different examiners.

The following points were analyzed:

1. Intra-rater reliability and inter-rater reliability of body surface area calculated by the body scanner software. This analysis is performed on the basis of 126 participants with two measurements.
2. Evaluation of available existing body surface area formulae compared to body scanner calculations. Comparisons were performed separately for males and females as well as different categories of body mass index (BMI).
3. Re-parametrization of body surface area formulae considering subgroups of sex and BMI.

For BMI, we chose the following categories in accordance with the World Health Organization (WHO) (2000):

Underweight: BMI < 18.5
Normal weight: 18.5 ≤ BMI < 25.0
Overweight: 25.0 ≤ BMI < 30.0
Obesity: 30.0 ≤ BMI

For subgroup analyses, we considered the three BMI categories: “normal weight”, “overweight”, and “obesity”. The BMI category “underweight” was omitted due to small sample size in our population-based study.

Prior to analysis, we checked for outliers by applying Grubbs outlier test (Grubbs 1969) with *α* = 1 %. This was performed as part of general preprocessing and epidemiologic quality control of the data (see also (Kuehnapfel et al. 2016) for details). Of note, no outliers were detected for the chosen significance level and for the measures considered here.

Agreement of repeated measurements of the 3D laser-based surface area and agreement of empirical formulae and corresponding 3D laser-based measurements were assessed by the overall concordance correlation coefficient (OCCC) (Barnhart et al. 2002). OCCC equals one if and only if the means and variances of the features are equal and the Pearson correlation is one. 95 % confidence intervals were obtained by estimating jackknife standard errors of the Fisher-transformed OCCC according to Efron (Efron 1981). To evaluate the OCCC, we chose the following categories in accordance with common statistical correlation classification (Dancey and Reidy 2011):

- OCCC ≥ 0.9: “excellent”.
- 0.9 > OCCC ≥ 0.7: “good”.
- 0.7 > OCCC ≥ 0.5: “moderate”.
- 0.5 > OCCC: “low”.

We also calculated relative bias and relative standard deviation of differences between formula-derived surface areas and 3D laser-based measurements. In detail, we determined the differences between formula-derived surface areas and estimated surface area by the body scanner. This difference is divided by the corresponding body scanner value to obtain relative differences. Then, means and standard deviations of these quantities are calculated.

The majority of empirical formulae can be traced back to a non-linear equation of the form

$$\text{Surface }=\text{ }{{\beta }_{0}}\times \text{ heigh}{{\text{t}}^{\beta }}_{\text{1}}\times \text{ weigh}{{\text{t}}^{\beta }}_{\text{2}}$$

or even more simple forms, e.g., as implemented in the formula of *Livingston and Lee*, which only considers body weight. Here, *β*_0_, *β*_1_, and *β*_2_ are parameters of the equation requiring estimation. This type of equation can easily be transformed into a linear equation by applying the logarithm.

We re-estimated the parameters of this model on the basis of the 1435 LIFE-Adult individuals for which classical anthropometry and body scanner data are available in parallel. These were grouped by sex and BMI to derive formulae for the corresponding subgroups. We also propose a universal formula with adjustment for sex and BMI. In this case, the formula reads as follows:

$$\text{Surface }=\text{ }{{\beta }_{0}}\times \text{ Heigh}{{\text{t}}^{\beta }}_{\text{1}}\times \text{ Weigh}{{\text{t}}^{\beta }}_{\text{2}}\times \text{ exp}({{\beta }_{\text{3}}}\times \text{ Sex})\text{ }\times \text{ exp}({{\beta }_{\text{4}}}\times \text{ BMI}).$$

Here, *Sex* is “1” if male and “0” if female.

All analyses were implemented and performed using the statistical software environment R 3.3.0 (http://www.r-project.org).

## Results

### Intra- and inter-rater reliability of body surface area assessment by 3D anthropometry

Reliability of 3D anthropometry was performed in a separate data set of 126 probands with repeated measurements.

On the basis of 67 individuals with two measurements of body surface area taken by the same investigator, we determined an excellent intra-rater OCCC of 0.998 [95% CI (0.997, 0.999)]. The absolute technical error of measurement (TEM) is 0.007 m^2^, while the relative TEM is 0.397 %.

For 59 individuals with two assessments of body surface area performed by two different investigators, we also determined an excellent inter-rater OCCC of 0.998 [95% CI (0.997, 0.999)]. The absolute TEM is 0.007 m^2^, and the relative TEM is 0.394 %.

### Validity of formulae for body surface area

Descriptive statistics of our study population can be found in Table 2. With help of the 3D derived body surface area, we evaluated the performance of 18 empirical formulae. Considered formulae are presented in Table 1.

**Table 2:** Distribution of study participants with respect to sex and BMI group

BMI group	Male			Female			Total		
	*N*	Age	Surface	*N*	Age	Surface	*N*	Age	Surface
Underweight	1	74	1.57 ± NA	6	40–57	1.54 ± 0.0834	7	40–74	1.54 ± 0.0770
Normal weight	154	25–78	1.83 ± 0.1176	248	25–77	1.65 ± 0.1126	402	25–78	1.72 ± 0.1437
Overweight	347	34–80	1.94 ± 0.1277	283	23–79	1.75 ± 0.1109	630	23–80	1.86 ± 0.1539
Obesity	185	31–77	2.09 ± 0.1552	211	33–78	1.93 ± 0.1609	396	31–78	2.00 ± 0.1774
Total	687	25–80	1.96 ± 0.1622	748	23–79	1.77 ± 0.1681	1435	23–80	1.86 ± 0.1907

Body scanner-derived surface given as mean ± standard deviation. Underweight individuals are excluded from subgroup analysis due to small sample size

**Fig. 1:**
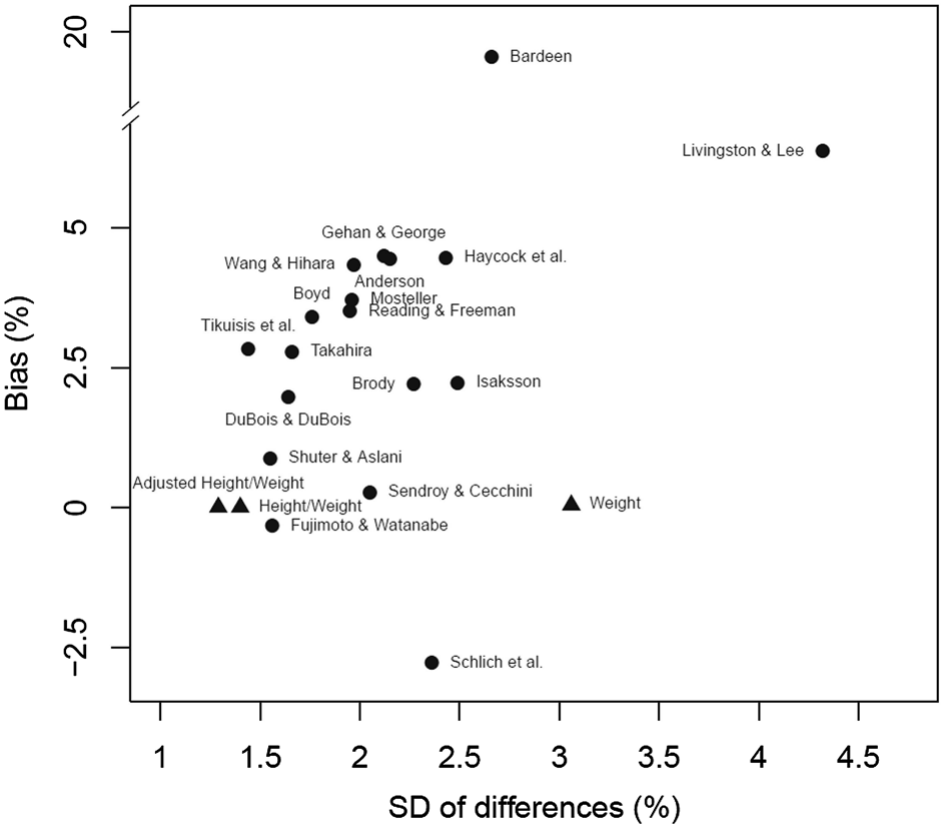
Accuracy of the 18 existing (*dots*) and 3 derived (*triangles*) formulae using 3D body scanner surface as reference. We present relative bias and relative standard deviation of the difference of the body scanner-derived surface areas and those derived by the empirical formulae

A complete overview of concordance results for the entire data set and all BMI scenarios is given in Table 3. OCCC for entire data set ranges from 0.409 (*Bardeen*) to 0.988 (*Fujimoto and Watanabe*). 15 out of 18 have OCCC values greater than 0.9. Best formulae are that of *Fujimoto and Watanabe* with an OCCC of 0.988 [95% CI (0.987, 0.990)] and that of *Shuter and Aslani* with an OCCC of 0.985 [95% CI (0.983, 0.986)], closely followed by the formula of *Sendroy and Cecchini* with an OCCC of 0.981 [95% CI (0.979, 0.982)]. Deviations from surface areas determined by body scanner are illustrated in Fig. 1. Here, we display the standard error and bias for each formula. Bland–Altman plots and corresponding mean differences and limits of agreement are provided in Supplementary Figs. 1 and 2. Statistics of the plots are provided in Supplementary Table 7. We observed a positive trend between average body surface area, and the difference of body scanner-derived and calculated body surface area for all empirical formulae, i.e., empirical formulae, tends to over-estimate body surface area for obese subjects but to under-estimate it for lean subjects.

**Table 3:** OCCCs and confidence intervals for comparison of 3D body scanner and formulae

Formula	All			Normalweight			Overweight			Obesity		
	OCCC	Lower 95% CI	Upper 95% CI	OCCC	Lower 95% CI	Upper 95% CI	OCCC	Lower 95% CI	Upper 95% CI	OCCC	Lower 95% CI	Upper 95% CI
Anderson	0.901	0.894	0.908	0.944	0.935	0.952	0.861	0.847	0.874	0.796	0.772	0.817
Bardeen	0.409	0.391	0.426	0.361	0.330	0.391	0.298	0.277	0.319	0.286	0.258	0.314
Boyd	0.936	0.931	0.941	0.958	0.950	0.964	0.906	0.895	0.915	0.875	0.859	0.890
Brody	0.955	0.952	0.959	0.984	0.981	0.987	0.949	0.943	0.955	0.883	0.868	0.897
DuBois and DuBois	0.969	0.967	0.972	0.965	0.959	0.970	0.950	0.944	0.955	0.959	0.952	0.965
Fujimoto and Watanabe	0.988	0.987	0.990	0.983	0.980	0.986	0.986	0.984	0.988	0.980	0.976	0.984
Gehan and George	0.900	0.893	0.907	0.942	0.932	0.950	0.859	0.845	0.872	0.796	0.772	0.818
Haycock et al.	0.896	0.889	0.903	0.953	0.945	0.960	0.861	0.847	0.874	0.775	0.750	0.798
Isaksson	0.954	0.949	0.958	0.936	0.922	0.948	0.937	0.929	0.944	0.928	0.918	0.937
Livingston and Lee	0.801	0.788	0.813	0.943	0.932	0.952	0.762	0.740	0.782	0.559	0.524	0.593
Mosteller	0.925	0.920	0.930	0.957	0.949	0.963	0.893	0.881	0.903	0.849	0.830	0.866
Reading and Freeman	0.931	0.926	0.936	0.962	0.955	0.967	0.902	0.891	0.911	0.858	0.840	0.874
Schlich et al.	0.946	0.941	0.950	0.937	0.927	0.946	0.933	0.925	0.941	0.911	0.895	0.925
Sendroy and Cecchini	0.981	0.979	0.982	0.980	0.976	0.983	0.975	0.972	0.977	0.968	0.963	0.972
Shuter and Aslani	0.985	0.983	0.986	0.987	0.984	0.989	0.977	0.973	0.980	0.974	0.969	0.978
Takahira	0.953	0.949	0.956	0.943	0.934	0.951	0.924	0.915	0.932	0.939	0.930	0.948
Tikuisis et al.	0.955	0.951	0.959	0.959	0.952	0.965	0.931	0.923	0.938	0.921	0.909	0.931
Wang and Hihara	0.907	0.901	0.913	0.938	0.929	0.947	0.866	0.852	0.878	0.821	0.799	0.840

Results of the entire data set and for all BMI scenarios

Repeating OCCC calculations for BMI (cf. Table 3) and sex subgroups (cf. Supplementary Tables 1, 2), we observed that either the formulae of *Fujimoto and Watanabe* or *Shuter and Aslani* achieves the highest OCCC values among the formulae.

The formula of *Bardeen* again showed worst results in all subgroup scenarios (maximum OCCC among subgroups is 0.390 in the subgroup of females). In the subgroup of obese individuals, the formula of *Livingston and Lee* also failed to achieve good agreement with an OCCC of 0.559 [95% CI (0.524, 0.593)]. The same applies for the formula of *Haycock et al.* in the subgroup of obese males with an OCCC of 0.675 [95% CI (0.630, 0.716)]. The other formulae yield excellent or good results for all subgroups considered.

### Re-estimated formulae for body surface area

Using data of body height and body weight and the body surface area derived by 3D anthropometry, we re-parameterized the standard form of surface area formula provided in the “Methods”. We also derived subgroup-specific formulae for males and females and different BMI categories. Parameter estimates and standard errors for all scenarios can be found in Table 4. We propose the general formula:

**Table 4:** Parameter estimates and corresponding standard errors within subgroups of sex and BMI

	All	Male	Female
All			
${{\hat{\beta }}_{0}}$_0_ (SE)	0.0151 (0.0005)	0.0091 (0.0005)	0.0110 (0.0007)
${{\hat{\beta }}_{1}}$ (SE)	0.5751 (0.0078)	0.6821 (0.0126)	0.6275 (0.0125)
${{\hat{\beta }}_{2}}$ (SE)	0.4259 (0.0022)	0.4144 (0.0033)	0.4386 (0.0028)
Normal weight			
${{\hat{\beta }}_{0}}$ (SE)	0.0110 (0.0009)	0.0070 (0.0011)	0.0105 (0.0011)
${{\hat{\beta }}_{1}}$ (SE)	0.6538 (0.0218)	0.7645 (0.0410)	0.6455 (0.0260)
${{\hat{\beta }}_{2}}$ (SE)	0.4069 (0.0085)	0.3777 (0.0172)	0.4292 (0.0098)
Overweight			
${{\hat{\beta }}_{0}}$ (SE)	0.0137 (0.0010)	0.0091 (0.0009)	0.0105 (0.0014)
${{\hat{\beta }}_{1}}$ (SE)	0.6030 (0.0221)	0.6770 (0.0277)	0.6494 (0.0352)
${{\hat{\beta }}_{2}}$ (SE)	0.4153 (0.0100)	0.4214 (0.0123)	0.4230 (0.0147)
Obesity			
${{\hat{\beta }}_{0}}$ (SE)	0.0211 (0.0017)	0.0126 (0.0016)	0.0122 (0.0017)
${{\hat{\beta }}_{1}}$ (SE)	0.4784 (0.0200)	0.5852 (0.0302)	0.5922 (0.0323)
${{\hat{\beta }}_{2}}$ (SE)	0.4618 (0.0079)	0.4528 (0.0103)	0.4568 (0.0102)

$$\text{Surface }=\text{ }0.0\text{151 }\times \text{Heigh}{{\text{t}}^{0.\text{5751}}}\times \text{ Weigh}{{\text{t}}^{0.\text{4259}}},$$

resulting in an adjusted *R* ^2^ of 0.9812.

Parameter estimates for the subgroups of sex and BMI differ slightly with a general trend that *β*_1_ (i.e. the weight of *Height* in the surface formula) is becoming smaller and *β*_2_ (i.e. the weight of *Weight* in the surface formula) is becoming larger for higher BMI. There are also moderate sex differences between parameters. A general formula adjusting for these (significant) factors is

$$\text{Surface }=\text{ }0.00\text{51 }\times \text{ Heigh}{{\text{t}}^{0.\text{8516}}}\times \text{ Weigh}{{\text{t}}^{0.\text{3262}}}\times \text{ exp}(-0.0\text{12}0\times \text{ Sex})\text{ }\times \text{ exp}(0.00\text{36}\times \text{ BMI}),$$

resulting in an adjusted *R* ^2^ of 0.9839. The standard errors for parameter estimates are given by: SE (${{\hat{\beta }}_{0}}$ = 0.0051) = 0.0006, SE (${{\hat{\beta }}_{1}}$ = 0.8516) = 0.0338, SE (${{\hat{\beta }}_{1}}$ = 0.3262) = 0.0166, SE (${{\hat{\beta }}_{3}}$ = −0.0120) = 0.0009, and SE (${{\hat{\beta }}_{4}}$ = 0.0036) = 0.0006.

As a simple alternative, we finally like to propose a formula which is based on body weight alone:

$$\text{Surface }=\text{ }0.\text{2}0\text{21}\times \text{ Weigh}{{\text{t}}^{0.\text{5}0\text{79}}},$$

resulting in an adjusted *R* ^2^ of 0.9106. The standard errors here are: SE (${{\hat{\beta }}_{0}}$ = 0.2021) = 0.0037 and SE (${{\hat{\beta }}_{1}}$ = 0.5079) = 0.0042.

For comparisons in-between new derived formulae, we chose the OCCC. The surface model with *Height* and *Weight* yields an OCCC of 0.990 [95% CI (0.989, 0.991)]. When using the extended model with adjustment by *Sex* and *BMI* one obtains an OCCC of 0.992 [95% CI (0.991, 0.993)]. The simple surface model with *Weight* as the only predictor yields an OCCC of 0.953 [95% CI (0.948, 0.957)]. For the entire data set, the surface formula with adjustment for *Sex* and *BMI* shows the best result of all formulae considered, but the difference to the non-adjusted formula is not significant (two-sided paired *t* test, *p* = 0.8737).

OCCC values of new surface formulae within subgroups are shown in Supplementary Tables 3, 4, and 5. We analyze, whether the subgroup-specific formulae provide any advantage compared to the general formula without adjustments. Indeed, subgroup-specific formulae yield higher OCCC throughout (cf. Supplementary Tables 3 to 5). The difference is significant for all comparisons except for the subgroup of males with normal weight (cf. Supplementary Table 6). On the other hand, the absolute differences are small in size reflected by the OCCCs of the general formula.

Finally, we also provide Bland–Altman plots for new formulae in Supplementary Figs. 3 and 4. The corresponding statistics are displayed in Supplementary Table 8. Almost zero correlation can be assumed for all new formulae.

## Discussion

Measurement of body surface area is important for several medical applications, e.g., drug dosage in cancer therapy and assessment of echocardiography or severity of skin lesions. A variety of formulae to calculate body surface area is available. Typically, body height and body weight are the only variables used when estimating surface area. 3D laser-based body scanning is a new method for precise measurement of body surface area. Instead of considering only two anthropometric measurements (body height and body weight), it uses a virtual twin consisting of about 500,000 datapoints determined by optical triangulation to estimate the surface. These detailed data allow a precise estimation of the body surface area. Although the technique is not available in general medical practice, it can be used to verify empirical formulae of body surface area as demonstrated in the present work.

3D body scanner VITUS XXL by Human Solutions with the analysis software ANTHROSCAN BASIS was used here. Of note, the software represents a “black box” and software version or devices may differ between studies. Comparability with other automated medical devices for anthropometric measurements is guaranteed by the DIN EN ISO 20685.

Daniell et al. (2012) already analyzed the performance of empirical formulae in contrast to 3D laser-based surface area. They also used Body Scanner Vitus XXL (laser class 1—safe with open eyes, Human Solutions, Kaiserslautern, Germany) but with another software CySlice v.3.4 (Headus, Perth, Australia). Sample size was comparable to the number of individuals considered in the present work. The age spectrum was 18–30 years, while we considered a more comprehensive age spectrum of 23–80 years. Moreover, we analyzed the impact of sex and BMI on the performance of empirical formulae and aimed at improving them by considering these factors and re-estimating the original parameters of the formulae. In the framework of a small feasibility study of repeated measurements, we also determined the intra- and inter-rater reliability of 3D laser-based surface measurements with excellent results. In a former work of our group (Kuehnapfel et al. 2016), we discovered that few 3D-derived anthropometric quantities are prone to measurement errors resulting in outliers. No such problems were detected for the body surface area studied here.

We performed a comprehensive analysis of the validity of existing formulae for surface estimation in our population-based cohort by comparing results with 3D body scans. Although there are numerous formulae estimating body surface area from body height and body weight, only three of them showed high validity even for the subgroups considered: *Fujimoto and Watanabe*, *Shuter and Aslani*, and *Sendroy and Cecchini*. Daniell et al. (2012) found that the formula of *Shuter and Aslani* performs best regarding accuracy and precision compared to the other formulae considered. The formula of *Fujimoto and Watanabe* was not considered there probably because it was developed for Asian populations. Interestingly, this formula also works very well for our population of Caucasian origin. Other formulae showed larger biases and variances with *Bardeen*’s formula yielding lowest accuracy, probably caused by a less suitable formula structure. The formula of *Livingston and Lee* showed the lowest precision among all formulae. These results are in accordance with Daniell et al. (2012). The widely used formulae by *DuBois and DuBois*, *Brody* or *Mosteller* were also in excellent agreement with the body scanner-derived surface estimates in the entire population. However, they are outperformed by the above-mentioned formulae. All empirical surface formulae tend to have lower OCCC values in the subgroup of overweight or obese individuals, except for *DuBois and DuBois*’, *Isaksson*’s, and *Takahira*’s formula with no detectable trend across BMI subgroups. In line with this observation, all empirical formulae considered show lower accuracy for extreme body surface areas as detected by Bland–Altman plots. A positive correlation was observed between average and difference of body scanner-derived and empirical body surface areas. This correlation was smallest for the empirical formulae of *Isaksson*, *Tikuisis et al.*, *Schlich et al.*, *Fujimoto and Watanabe*, *Shuter and Aslani*, *DuBois and DuBois*, *Takahira*, and *Sendroy and Cecchini*.

We used our large data set to re-parameterize body surface area formulae. For this purpose, we adopted the most frequently used formula structure calculating surface area via a power function of body height and body weight [e.g., (Schlich et al. 2010; Wang and Hihara 2004; Tikuisis et al. 2001)]. Due to their good performance, there was no reason to change the mathematical structure of the model. We estimated parameters for the entire data set and for subgroups of sex and BMI. An extended model with adjustment for sex and BMI as well as a simple model with body weight as the only predictor were also proposed. Comparing models by adjusted *R* ^2^, the model with adjustment for sex and BMI shows the best compromise between model complexity and fit considering the whole population.

We also re-parameterized the general surface formula for the subgroups considered. Differences of surface formulae of subgroups and the general surface formula were significant for all subgroups except for normal-weight males. Subgroup-specific formulae also outperform the adjusted surface formula in the subgroups of normal-weight individuals and the sex-specific BMI subgroups.

All new formulae show marginal negative correlation of average and difference regarding body scanner-derived surface area. The surface formula with adjustment for sex and BMI yielded the smallest correlation within all new derived formulae.

Regarding generalizability, we observed good agreement of empirical formulae with body scanner-derived surface area across sex and BMI subgroups indicating that the formulae are relatively robust with respect to extreme builds. This points towards general applicability of the formulae also to other ethnicities. The latter is also supported by the observation that the formula of *Fujimoto and Watanabe* shows excellent performance in our European population, although it was developed in an Asian population.

Our study has some limitations: although our sample size was relatively high, allocation number of the subgroup of males with normal weight was relatively small. The subgroup of underweight individuals was essentially not present in our population-based sample, where extreme builds are less frequent. Furthermore, body surface area by 3D body scanner was considered as valid assessment of body surface area throughout our analyses. Although the body scan provides a much higher degree of details, only parts of the body which are “visible” to the four lasers can be exactly mapped and measured. Finally, we derived formulae for surface estimation on the basis of one particular model structure.

## Conclusion

We observed an excellent reliability of 3D laser-based body surface assessments. Empirical formulae of body surface area proposed in the literature could be verified on the basis of a large population-based cohort. We could show that the formulae of Fujimoto and Watanabe, Shuter and Aslani, and Sendroy and Cecchini give excellent results even for the subgroups of sex and BMI considered here. Results could be refined by a modified formula, including sex and BMI subgroup as covariables but the improvement is of small extent.

## Electronic supplementary material

Below is the link to the electronic supplementary material.

Supplementary material 1 (XLSX 44 KB)

Supplementary material 2 (PNG 179 KB)

Supplementary material 3 (PNG 187 KB)

Supplementary material 4 (PNG 169 KB)

Supplementary material 5 (PNG 98 KB)

## Abbreviations

BMI: Body mass index
LIFE: Leipzig Research Center for Civilization Diseases
OCCC: Overall concordance correlation coefficient
