# Supplementary figures and images for: Body surface assessment with 3D laser-based anthropometry: reliability, validation, and improvement of empirical surface formulae
Source: Eur J Appl Physiol. 2017 Jan 27;117(2):371–80. doi: 10.1007/s00421-016-3525-5 (PMC5313586; doi:10.1007/s00421-016-3525-5)

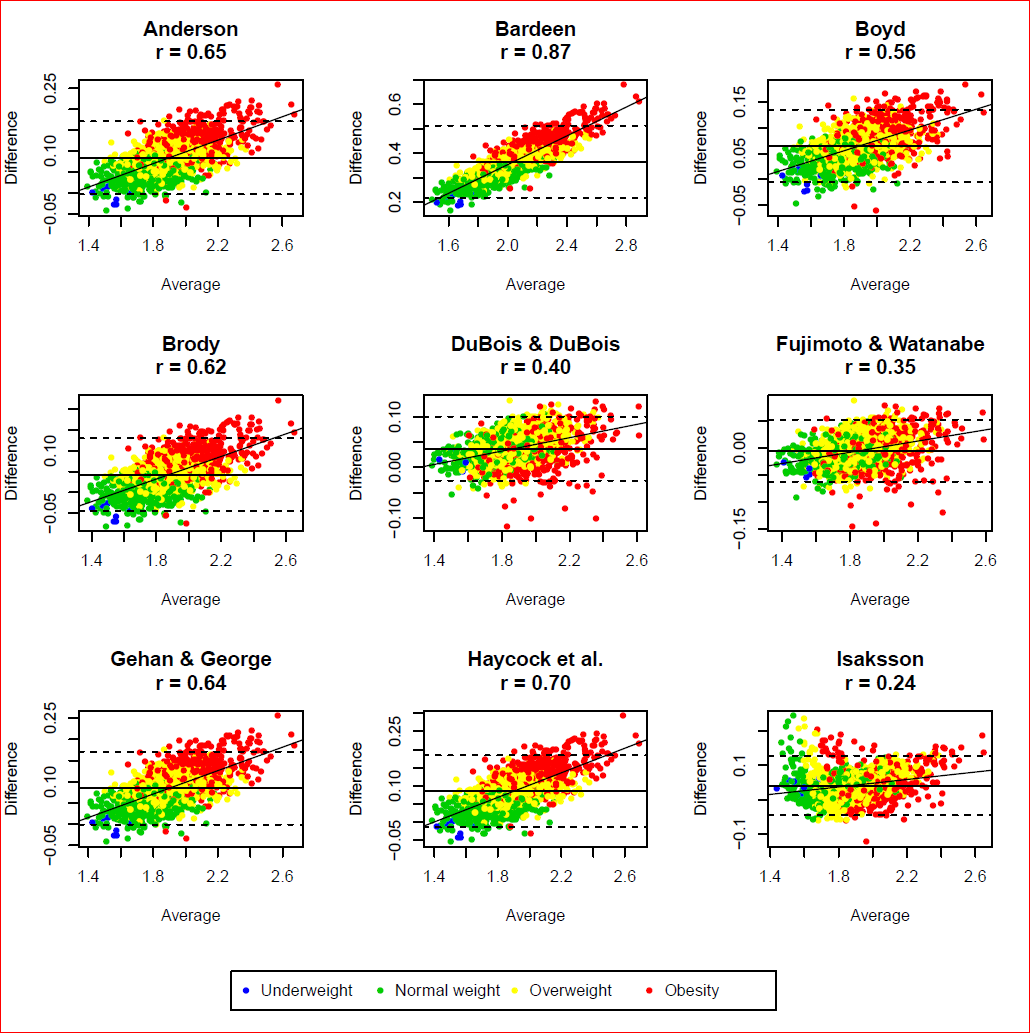

Supplement: Supplementary file 2 — Supplementary material 2 (PNG 179 KB) [file 421_2016_3525_MOESM2_ESM.png]

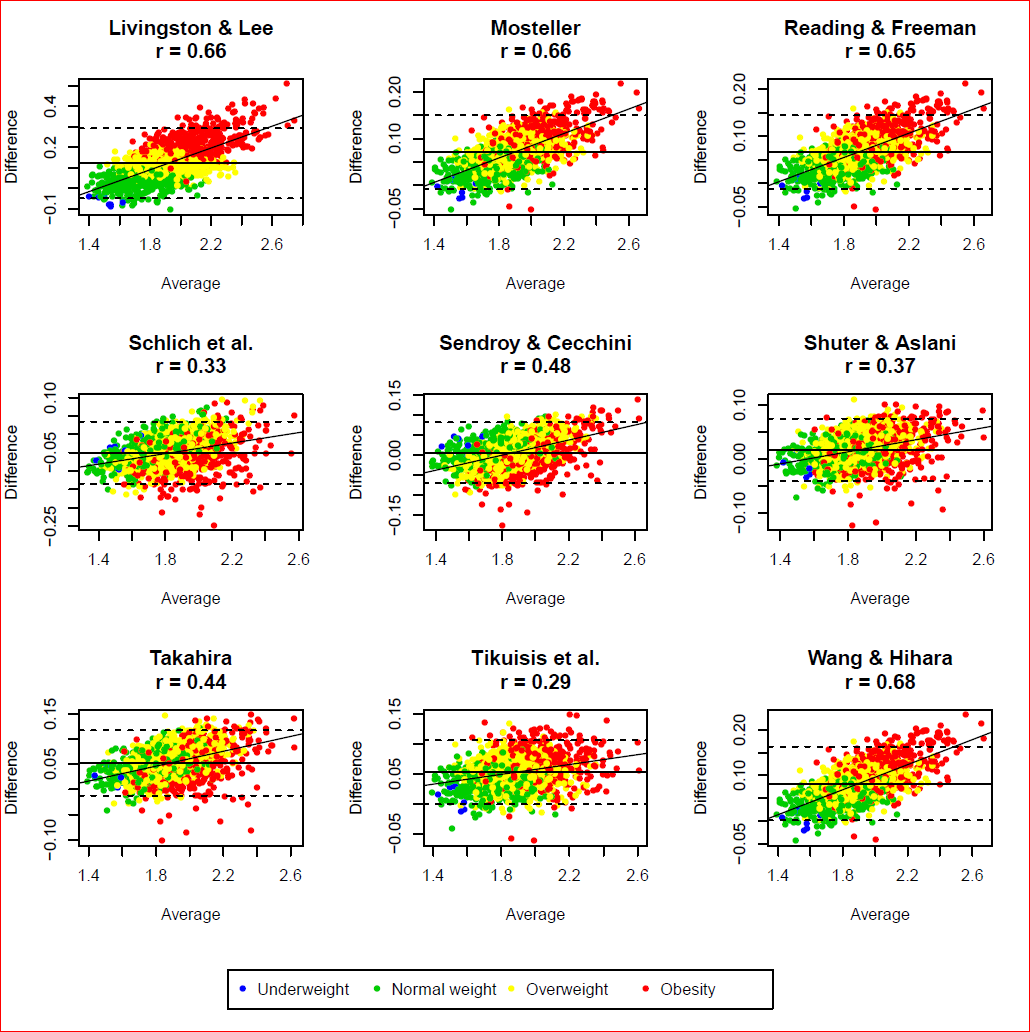

Supplement: Supplementary file 3 — Supplementary material 3 (PNG 187 KB) [file 421_2016_3525_MOESM3_ESM.png]

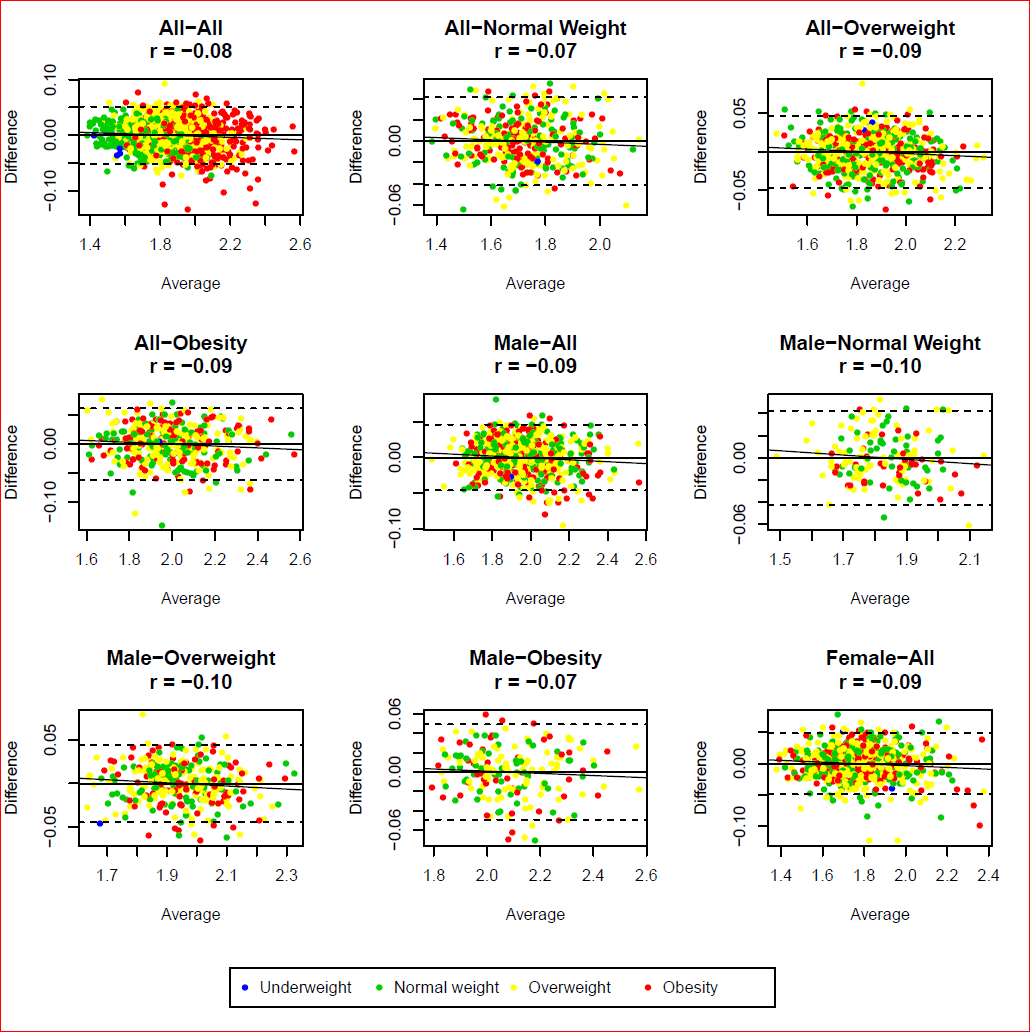

Supplement: Supplementary file 4 — Supplementary material 4 (PNG 169 KB) [file 421_2016_3525_MOESM4_ESM.png]

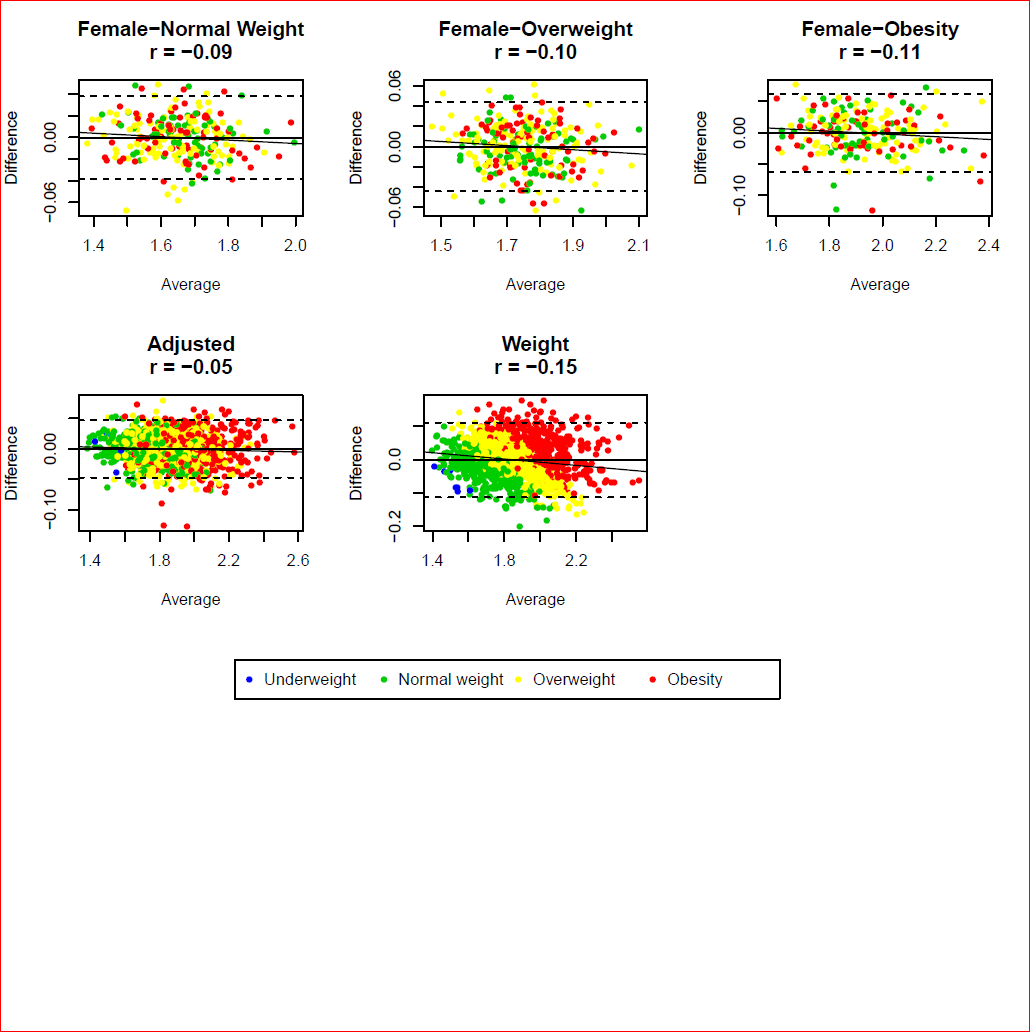

Supplement: Supplementary file 5 — Supplementary material 5 (PNG 98 KB) [file 421_2016_3525_MOESM5_ESM.png]
